# Endoscopic cervical open-door laminoplasty: A case and technical report

**DOI:** 10.1016/j.ijscr.2025.111645

**Published:** 2025-07-12

**Authors:** Shu Nakamura, Zenya Ito, Motohide Shibayama

**Affiliations:** a Department of Orthopedic Surgery, Aichi Spine Hospital, 31-1 Kamiike, Goroumaru, Inuyama-shi, Aichi 484-0066, Japan

**Keywords:** Micro-endoscope, mini-plate, Myelopathy, Minimally invasive, Case report

## Abstract

**Introduction:**

Open-door laminoplasty (ODL) has been widely used to treat multilevel cervical spondylotic myelopathy. Recently, minimally invasive laminectomy using an endoscope has also been performed. However, postoperative MRI images often show uneven decompression. ODL under endoscopic guidance is a difficult task, and there are very few reports of such procedures.

**Presentation of case:**

A 73-year-old male. The patient presented with numbness in both upper limbs and gait disturbance, and spinal stenosis was observed at the C5–7 levels. To perform micro-endoscopic ODL in narrow spaces and by a single surgeon, procedure and fixation tools were developed. Sufficient spinal canal expansion without residual partial stenosis was achieved, leading to the improvement of myelopathy symptoms. Muscle atrophy was minimal, and bone fusion was accomplished.

**Discussion:**

This minimally invasive method was more effortless and reliable than ODL with a full-endoscope and suture anchor. The fixation was considered sufficiently stable.

**Conclusions:**

While this method may require additional knowledge accumulation in the future, it is considered to be a viable option.

## Highlights

• To perform Micro-endoscopic ODL in narrow spaces and by a single surgeon, procedure and fixation tools were developed.
• This surgery was minimally invasive, and both imaging and clinical outcomes were favorable.
• ODL using full-endoscope and suture anchors is fundamentally difficult to prevent lamina closure.
• This Micro-endoscopic ODL offers more reliable fixation and is easier.

## 1 Introduction

Open-door laminoplasty (ODL) has been widely used to treat multilevel cervical spondylotic myelopathy, and it remains one of the mainstream surgical techniques even now, nearly 50 years after it was first reported in 1977 [1]. ODL can sufficiently enlarge the spinal canal while reconstructing the posterior structures. Although the clinical results of this technique were reported to be favorable [2], this procedure involves a large skin incision, extensively detaching the muscles outward beyond the vertebral arch, and continued strong lateral retraction, which causes considerable muscle damage [3]. Postoperative axial pain can occur [3] and leave a large, ugly, depressed wound.

On the other hand, endoscopic posterior spinal canal decompression significantly reduces skin incision and muscle compression. But the enlargement of the spinal canal is limited, and it has been reported to be applicable to one or two intervertebral levels [4]. This procedure involves partial laminotomy and ligamentum flavum removal around the interlaminar space to avoid laminectomy [4]. However, postoperative MRI images often show uneven decompression, leaving areas that are not sufficiently decompressed. ODL tends to provide sufficient spinal canal expansion without residual partial stenosis across the affected area, resulting in more desirable imaging outcomes. In laminoplasty, a group of tissues including the epidural soft tissue, the ligamentum flavum, and the lamina is preserved and prevents the proliferation of epidural scar tissue [5] referred to as the laminectomy membrane [6]. On the other hand, in endoscopic decompression surgery, the bone and the yellow ligament are mostly removed, so the scar tissue proliferates behind the dura, leaving only a small space [5].

Performing ODL under endoscopy to combine the advantages of both techniques presents significant challenges. Typically, endoscopic surgery involves resection and removal, without true reconstruction. In this study, we performed endoscopic cervical ODL using a micro-endoscope to achieve sufficient decompression across multiple intervertebral levels with a minimally invasive approach, and we report our findings.

This case was reported following SCARE checklist.

## 2 Case presentation

### 2.1 History and examination

A 73-year-old male. For 10 years, he had numbness and pain in both upper extremities, which gradually worsened. Recently, he has also developed fine motor skill impairment, marked decrease in walking speed, walking limitation of less than 100 m, and urinary dysfunction. Deep tendon reflexes in both upper and lower limbs are mildly exaggerated.

**Fig. 1:**
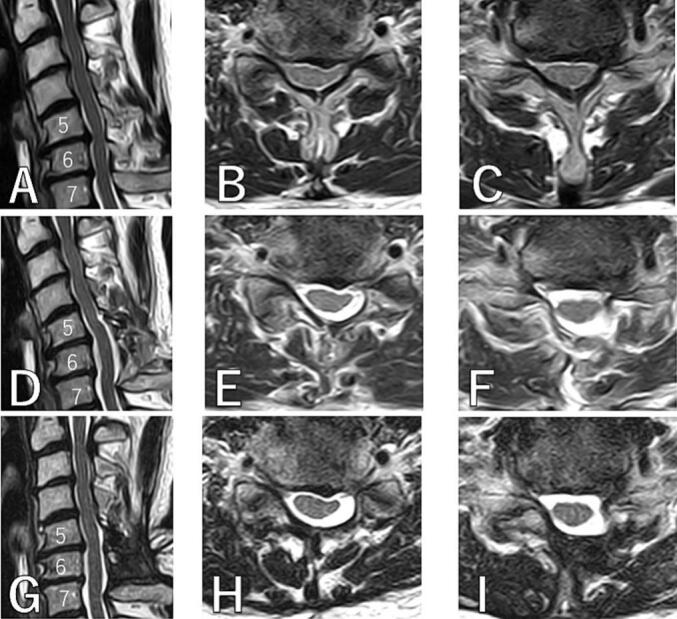
MR images. A, B, C: preoperative. D, E, F: postoperative. G, H, I: 2 years postoperative. A, D, G: sagittal. B, E, H: axial at C5–6. C, F, I: axial at C6–7.

MR images revealed cervical stenosis at the C5–6 and C6–7 intervertebral levels due to decreased disc height, disc protrusion, and ligamentum flavum hypertrophy (Fig. 1A-C).

### 2.2 Surgical procedure

Under general anesthesia, the patient was placed prone on a spine table. Without an assistant, the surgeon stood on the patient's left side. A 24 mm longitudinal skin incision was made at the dorsal center of the C6 level (Fig. 3A). The C6 spinous process was exposed.

A 16 mm outer tube was inserted using the serial dilator, and a micro-endoscope was positioned. The caudal side of the C5 lamina was exposed and resected using a diamond burr (Fig. 4A). Next, the cranial side of the C7 lamina was exposed and resected (Fig. 4B). The ligamentum flavum was not removed in either intervertebra. The left side of the C6 lamina was cut (Fig. 4C), and a hole was drilled medially from the cut end using a 2 mm diamond burr (Fig. 4D). The edge of the bone-cutting end cracked during screw insertion (Fig. 4E), and the screw could not be inserted. A bone gutter for the hinge was made on the right side of the C6 lamina (Fig. 4F). The lamina was expanded with the Kerrison punch (Fig. 4G). After drilling a hole lateral to the cut end of the lamina with a 2 mm diamond burr (Fig. 4H), a screw (2.6 mm in diameter, 7 mm in length) was inserted. A small gap (asterisk) was intentionally left between the bone surface and the screw head (Figs. 3E, 4I).

After removing the outer tube and inserting the retractor, the micro-endoscope was placed on the opposite side using a custom-made scope holder (Fig. 3B). The plate (lateral hole style, standard mouse) of Centerpiece (Medtronic, USA)was cut so that half of the screw hole remains on both the lamina side and the lateral mass side (Fig. 3C, D). The plate was attached to the plate holder, which was held in the right hand, and a curved curette was held in the left hand. While expanding the lamina with the curette, the lamina retaining part of the plate was fitted around the cut end of the lamina (Figs. 3F, 4J). Next, the lateral mass side of the plate was placed on the cut edge of the lateral mass while fitting the half screw hole under the previously inserted screw head (Figs. 3G, 4K). The stability of both the plate and the lamina was confirmed (Fig. 4L).

The drainage tube was removed 48 h postoperatively. As with a conventional laminoplasty, the patient wore a soft cervical collar for one week, and large neck movements were avoided for six weeks. This work has been reported in line with the SCARE criteria [7].

### 2.3 Postoperative images

**Fig. 2:**
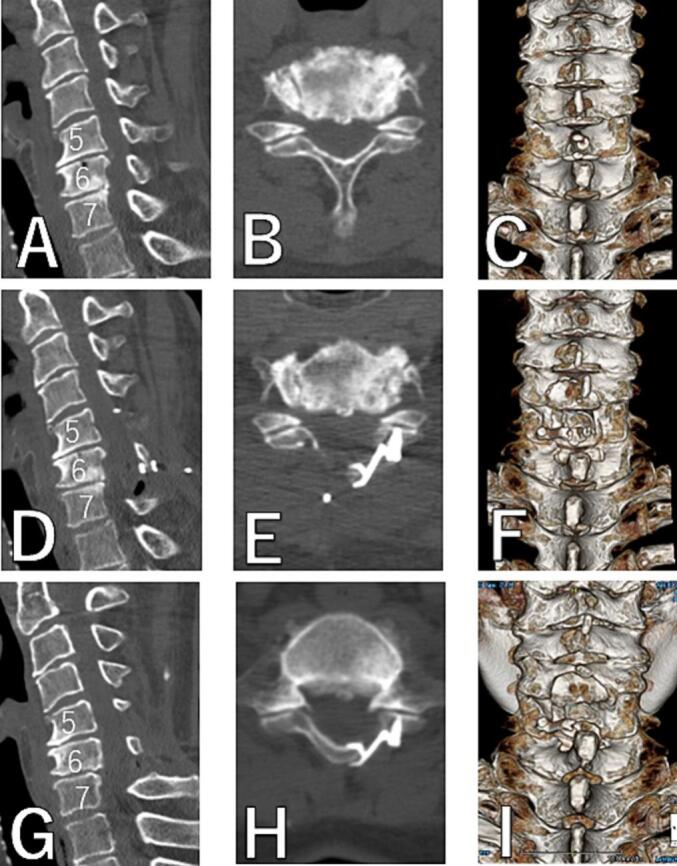
CT. A, B, C: preoperative. D, E, F: postoperative. G, H, I: 2 years postoperative. A, D, G: sagittal. B, E, H: axial at C5. C, F, I: 3D.

The spinal canal expanded sufficiently at the C5-6-7 levels in postoperative MR images (Fig. 1D-F). In postoperative CT images, it can be seen that the cut end of the lamina was slightly chipped as previously mentioned, causing limited contact between the cut end of the lamina and the plate, but the lamina's expansion was still maintained (Fig. 2D-F).

Two years postoperative MR images showed that the spinal canal at the C5–6-7 levels was still adequately wide, and muscle atrophy was only mild (Fig. 1G-I). Two years postoperative CT images showed that the plate had not displaced at all, the lamina had remained expanded, and bone fusion was observed at the lamina hinge (Fig. 2G-I).

### 2.4 Clinical result

The amount of blood loss was 60 ml. The patient ambulated without any problems 3 h postoperatively. The postoperative wound pain VAS was 20 on the day of surgery and dropped to 0 by the following morning, indicating minimal wound pain. There were no complications related to the surgery. The postoperative follow-up period lasted for 2 years. Numbness and pain in the upper limbs were reduced to only the fingertips of the 3rd and 4th fingers. Clumsy hands, gait disturbance, and urinary dysfunction disappeared. Clinical outcomes (preoperative to final) were neck pain VAS (8 to 0), arm pain VAS (8 to 4), The Japanese Orthopedic Association (JOA) score for cervical myelopathy revised 17-point method (11 to 16), and the JOA score improvement rate was 83 %.

## 3 Discussion

### 3.1 The road to endoscopic ODL

First, in order to perform minimally invasive ODL using an endoscope, we devised a method using full-endoscope in 2017. Partial laminotomy, lamina cutting and hinge creation were performed under full-endoscope via bilateral posterior approach. Suture anchors fixed to the base of the spinous process and the lateral side of the lateral mass via a lateral approach (Fig. 5A). While maintaining the opening of lamina by pulling the thread, another thread was tied outside the skin incision, and the knot was tightened using a knot pusher to complete the fixation (Fig. 5B-D). In Case, laminoplasty was performed on C5 by decompressing C4–6. But the expansion reverted (Fig. 5E, F). However, the symptoms improved. This method is difficult to perform alone and requires two experienced surgeons. Additionally, ensuring the maintenance of the expansion is difficult. However, similar methods, though slightly different, have been reported by Zhu et al. [8] in 2022. It is not impossible, but in these full-endoscopic situations where the entire surgical field cannot be observed at a glance during suturing, it is difficult to tighten the thread to the appropriate length while using a knot pusher to push and fasten the wobbling thread. Therefore, it was fundamentally difficult to reduce lamina closure, and we decided to use a miniplate.

Next, we devised and performed this method in 2022. Although it is not an easy method, it was sufficiently achievable using accustomed techniques. The stability of the expansion was achieved, and bone fusion occurred with no issues in the follow-up.

### 3.2 Stability

**Fig. 3:**
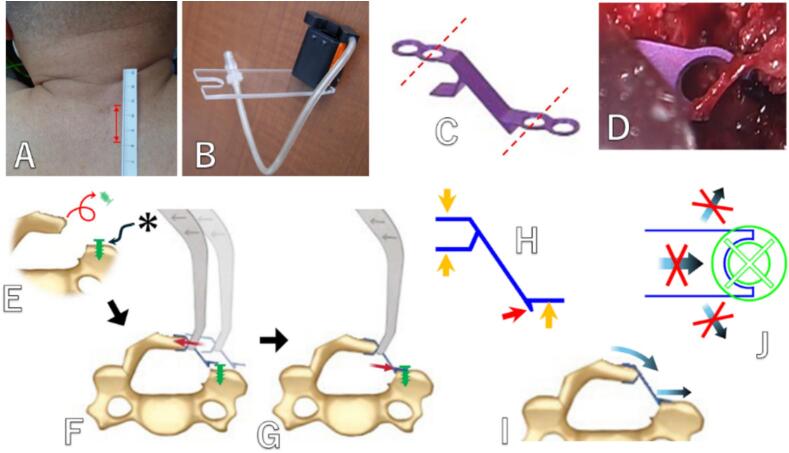
Endoscopic cervical ODL using a micro-endoscope. A: 24 mm longitudinal skin incision. B: custom-made scope holder. C: The plate of Centerpiece (Medtronic, USA) was cut so that half of the screw hole remains on both the lamina side and the lateral mass side. D: Half of the screw hole on the lateral mass side. E: Schematic of the surgery procedures. A screw was inserted at the lateral mass. A small gap (asterisk) was left between the bone surface and the screw head. F: The plate's lamina retaining part was fitted around the cut end of the lamina while expanding it. G: The lateral mass side of the plate was placed on the cut edge of the lateral mass while fitting the half screw hole under the previously inserted screw head. H: Three parts of the plate indicated by the orange arrow are sufficiently long, but the part indicated by the red arrow is short due to the presence of nerves. I: Therefore, there is a risk of the plate being pushed outward and displacement due to the closing force of the lamina. J: The screw inserted into the lateral mass safeguards against this risk, and there are virtually no other risks to displacement. (For interpretation of the references to colour in this figure legend, the reader is referred to the web version of this article.)

There were concerns about the stability of the fixation, but we have previously not encountered problems despite not performing screw fixation at the lamina retaining part of the plate. Additionally, there have been reports of ODL performed using an HA spacer without fastening it [9,10], and these reports indicate that there have been a few cases of spacer displacement (0.2–1.7 %). It is believed that the elasticity of the hinge and the tension of the surrounding muscles provide stability. Three parts of the plate indicated by the orange arrow in Fig. 3H are sufficiently long, but the part indicated by the red arrow is short due to the presence of nerves. Therefore, there is a risk of the plate being pushed outward and displacement due to the closing force of the lamina (Fig. 3I). This risk is prevented by the screw inserted into the lateral mass (Fig. 3J), and it is considered that there are almost no other risks of displacement. Although we were not able to insert the planned screw on the lamina side in this case, the results showed that there was no problem even without it. This method is very simple, but it is believed to meet the necessary and sufficient conditions for fixation stability.

**Fig. 4:**
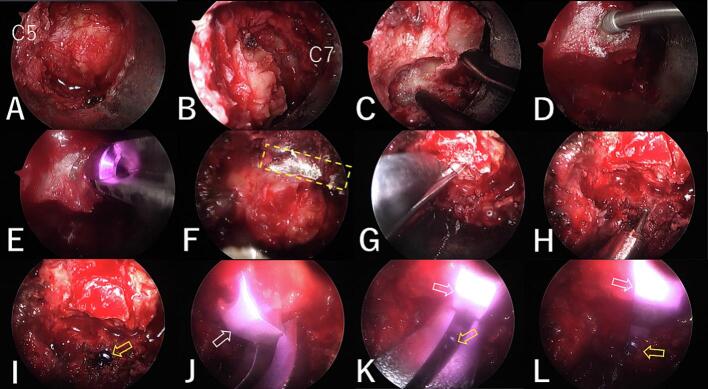
Endoscopy images. In the figure, the cranial side is on the left, the caudal side on the right, the left side is at the bottom, and the right side is at the top. A: The caudal side of the C5 lamina was resected. B: The cranial side of the C7 lamina was resected. C: The left side of the C6 lamina was cut. D: A hole was drilled medially from the cut end. E: The edge of the bone-cutting end cracked during screw insertion. F: A bone gutter (yellow dashed line) for the hinge was made on the right side of the C6 lamina. G: The lamina was expanded with the Kerrison punch. H: A hole was drilled laterally from the cut end. I: A screw (yellow arrow) was inserted with a gap between the lamina and the screw head. J: The lamina retaining part of the plate (white arrow) was fitted around the cut end of the lamina. K: The lateral mass side of the plate was placed on the cut edge of the lateral mass while fitting the half screw hole under the previously inserted screw head. L: The plate holder was taken out. (For interpretation of the references to colour in this figure legend, the reader is referred to the web version of this article.)

**Fig. 5:**
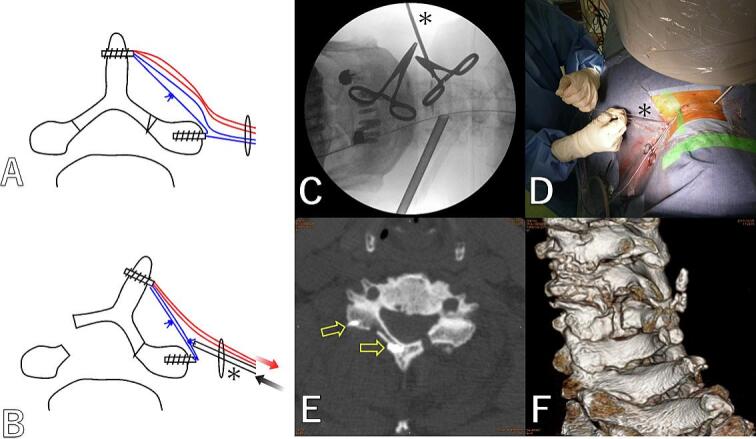
Minimally invasive ODL under full-endoscope. A: Suture anchors fixed to the base of the spinous process and the lateral side of the lateral mass. B: While maintaining the opening of lamina by pulling the thread, another thread was tied outside the skin incision, and the knot was tightened using a knot pusher (asterisk) to complete the fixation. C: Fluoroscopic image. D: While maintaining the opening of lamina by pulling the thread, the knot was tightened using a knot pusher to complete the fixation. E: Postoperative CT axial image at C5. The expansion of the lamina reverted. (Anchor: yellow arrow) F: CT 3D image. Laminoplasty was performed on C5 by decompressing C4–6. (For interpretation of the references to colour in this figure legend, the reader is referred to the web version of this article.)

### 3.3 Operability

While the minimum workspace is secured using a retractor and the field of view is ensured with an endoscope, it was still considered difficult to secure enough space and visibility to simultaneously hold the plate and insert the screw. Therefore, this technique introduced a novel approach to avoid simultaneous handling by first placing the screw and subsequently positioning a plate with a halved screw hole beneath the screw head. It was confirmed through this practice that this method is not difficult to execute.

### 3.4 Choice of endoscope

It will be also possible to perform this method using UBE, but ODL requires extensive detachment over the lamina, which is not a strong suit of UBE. Micro-endoscopic surgery is performed in an air medium, allowing for rapid dissection using a monopolar electrocautery device. Additionally, in UBE, there are concerns about the difficulty of securing the visual field due to extensive bleeding and the effects of prolonged water pressure on the spinal cord. Gong et al. reported ODL using UBE [11] in 2024, but after the hinge and cuts are made in UBE, plate fixation is performed openly through a large skin incision, so it is not entirely done under endoscopy. Our method completes the procedure minimally invasively, including plate fixation, entirely under endoscope.

### 3.5 Invasiveness

Compared to conventional ODL, most of the surgical procedures are performed through a 16 mm outer tube, resulting in less muscle compression. The muscle detachment on the lateral mass side is also minimized. It is possible to preserve the epidural tissue, ligamentum flavum, and lamina, which, as mentioned earlier, has benefits, and it is believed to reduce the risk of epidural bleeding and dural injury.

### 3.6 Limitations

The development of this method was based on several preliminary cases. However, only one case has been implemented in the main study, and more cases are necessary to build upon these results.

## 4 Conclusion

This method for cervical spondylotic myelopathy was found to be safe and effective, and the spinal canal enlargement was achieved with minimal invasiveness. Using this method and gaining more experience could potentially make it a viable option for treating this disease, as we believe based on this result.

## Author contribution

The first author contributed to all aspects of the paper, including study concept and design, data collection, data analysis and interpretation, and writing the paper.

## Consent

Written informed consent was obtained from the patient for publication of this case report and accompanying images. A copy of the written consent is available for review by the Editor-in-Chief of this journal on request.

## Ethical approval

The study approval was obtained from Aichi Spine Hospital institutional review board (NO. IR18304).

## Guarantor

Shu Nakamura.

## Research registration number

Not applicable.

## Funding

This study received no specific grant from any funding sources.

## Conflict of interest statement

The authors have no relevant financial or non-financial interests to disclose.
